# Hyperspectral Imaging for Tissue Classification after Advanced Stage Ovarian Cancer Surgery—A Pilot Study

**DOI:** 10.3390/cancers14061422

**Published:** 2022-03-10

**Authors:** Sharline M. van Vliet-Pérez, Nick J. van de Berg, Francesca Manni, Marco Lai, Lucia Rijstenberg, Benno H. W. Hendriks, Jenny Dankelman, Patricia C. Ewing-Graham, Gatske M. Nieuwenhuyzen-de Boer, Heleen J. van Beekhuizen

**Affiliations:** 1 Department of Biomechanical Engineering, Delft University of Technology, 2628 CD Delft, The Netherlands; n.vandeberg@erasmusmc.nl (N.J.v.d.B.); b.h.w.hendriks@tudelft.nl (B.H.W.H.); j.dankelman@tudelft.nl (J.D.); 2 Department of Radiotherapy, Erasmus MC Cancer Institute, University Medical Center Rotterdam, 3015 GD Rotterdam, The Netherlands; 3 Department of Gynecological Oncology, Erasmus MC Cancer Institute, University Medical Center Rotterdam, 3015 GD Rotterdam, The Netherlands; g.nieuwenhuyzen-deboer@erasmusmc.nl (G.M.N.-d.B.); h.vanbeekhuizen@erasmusmc.nl (H.J.v.B.); 4 Department of Electrical Engineering, Eindhoven University of Technology, 5612 AZ Eindhoven, The Netherlands; f.manni@tue.nl (F.M.); m.lai@tue.nl (M.L.); 5 Department of Pathology, Erasmus University Medical Center Rotterdam, 3015 GD Rotterdam, The Netherlands; l.rijstenberg@erasmusmc.nl (L.R.); p.ewing@erasmusmc.nl (P.C.E.-G.); 6 Department of Gynecology, Albert Schweitzer Hospital, 3318 AT Dordrecht, The Netherlands

**Keywords:** hyperspectral imaging, ovarian epithelial carcinoma, cytoreduction surgical procedure, support vector machine, classification

## Abstract

**Simple Summary:**

Survival of ovarian cancer patients largely relies on the surgical removal of all cancer cells. To achieve this, good vision is crucial. In this study, we evaluate the ability of hyperspectral imaging to detect ovarian cancer. Images of surgically removed tissue samples of 11 patients were taken and compared to histopathology in order to train machine learning software. For training purposes, only healthy tissues and tissues with high tumor cell content (>50%) were included. In total, 26 tissue samples and 26,446 data points of 10 patients were included. Tissue classification as either tumorous or healthy was evaluated by leave-one-out cross-validation. This resulted in a power of 0.83, a sensitivity of 0.81, a specificity of 0.70 and a Matthew’s correlation coefficient of 0.41. To conclude, this study shows that hyperspectral imaging can be used to recognize ovarian cancer. In the future, the technique may enable real-time image guidance during surgery.

**Abstract:**

The most important prognostic factor for the survival of advanced-stage epithelial ovarian cancer (EOC) is the completeness of cytoreductive surgery (CRS). Therefore, an intraoperative technique to detect microscopic tumors would be of great value. The aim of this pilot study is to assess the feasibility of near-infrared hyperspectral imaging (HSI) for EOC detection in ex vivo tissue samples. Images were collected during CRS in 11 patients in the wavelength range of 665–975 nm, and processed by calibration, normalization, and noise filtering. A linear support vector machine (SVM) was employed to classify healthy and tumorous tissue (defined as >50% tumor cells). Classifier performance was evaluated using leave-one-out cross-validation. Images of 26 tissue samples from 10 patients were included, containing 26,446 data points that were matched to histopathology. Tumorous tissue could be classified with an area under the curve of 0.83, a sensitivity of 0.81, a specificity of 0.70, and Matthew’s correlation coefficient of 0.41. This study paves the way to in vivo and intraoperative use of HSI during CRS. Hyperspectral imaging can scan a whole tissue surface in a fast and non-contact way. Our pilot study demonstrates that HSI and SVM learning can be used to discriminate EOC from surrounding tissue.

## 1. Introduction

Ovarian cancer is the number eight cause of cancer-related mortality in women around the world, with an incidence of 314,000 and mortality of 207,000 in 2020 [1]. The International Federation of Gynecology and Obstetrics (FIGO) staging system defines the stages of ovarian cancer according to the extension of the tumor, the spread of the lymph nodes and the spread to distant sites [2]. Since early warning signs are often vague or missing, ovarian cancer is often detected in late stages (FIGO IIB–IV) [3]. 

The standard therapy for advanced-stage ovarian cancer is complete cytoreductive surgery (CRS) of all visible tumors and six cycles of chemotherapy [3,4]. Complete resection of all macroscopic disease is the strongest independent variable in predicting overall survival. However, even after complete CRS and removal of all visible and palpable tumors, women with advanced epithelial ovarian cancer can experience recurrence, possibly as a result of microscopic residual tumors. If microscopic, invisible and non-palpable tumors could be detected during surgery, progression-free and overall survival could increase [5]. Furthermore, when no microscopic tumor is detected, healthy tissue can be spared, thus potentially shortening the duration of surgery and recovery.

There are several intraoperative visualization techniques in use or development for the detection of epithelial ovarian cancer (EOC) metastasis: the most commonly used are cryosection and immunofluorescence. Cryosection is a widely used intraoperative diagnostic technique in which a small piece of the suspicious tissue is frozen, sliced and microscopically examined by the pathologist [6]. Disadvantages of this technique are the limited section size, the need to excise the tissue and the required processing time during surgery. During immunofluorescence, agents such as folate-FITC, 5-aminolevulinic acid, indocyanine green, and OTL38 are intravenously injected or taken orally by the patient. These fluorescent agents accumulate in malignant tumor cells and can be intraoperatively detected by an imaging system. The agents can produce mild adverse effects, such as gastrointestinal disorder, nausea, vomiting, effusion, loss of appetite, diarrhea and abdominal pain [7,8,9,10,11].

As an alternative, hyperspectral imaging (HSI) is a non-invasive, non-contact, and label-free imaging technique with the potential of detecting malignant tissue. HSI captures multiple images of the underlying tissue in contiguous spectral bands. With this data, a 3D hyperspectral (HS) cube can be built, containing spatial information in two dimensions and spectral information in one dimension [12]. The reflectance measured is related to the absorption and scattering properties of tissue [13]. This spectral signature of the underlying tissue can be used for the classification of both tumor and non-tumor tissue. HSI already shows promising results in other oncological fields [14,15,16,17,18,19,20].

The aim of this study is to evaluate and prove the potential and feasibility of HSI for EOC detection. To the best of our knowledge, studies involving HSI for EOC detection are limited. Hereby, we employ HSI for EOC and healthy tissue classification using ex vivo samples collected after surgery and their corresponding histopathological annotation used as ground truth. The ex vivo approach can form a basis for further in vivo studies where data is collected and examined intraoperatively. In this context, HSI is a novel aided tool to enhance the vision of the surgeon, guiding the tumor resection and reducing recurrence of cancer and second re-operations.

## 2. Materials and Methods

### 2.1. Participants

The recruitment of participants took place at Erasmus University Medical Center, Rotterdam. Patients of 18 years and older with known or high clinical suspicion of primary ovarian cancer, planned for either primary or interval CRS, and who were able and willing to comply with the study procedure, were enrolled into the study during May–October 2020. All participants signed an informed consent before any study-related procedure was performed. The study was carried out according to the standards outlined in the Declaration of Helsinki. All procedures involving patients have been approved by the Medical Ethical Committee of Erasmus Medical Center Rotterdam in the Netherlands (trial protocol MEC-2020-0167).

### 2.2. Instrumentation

**Figure 1:**
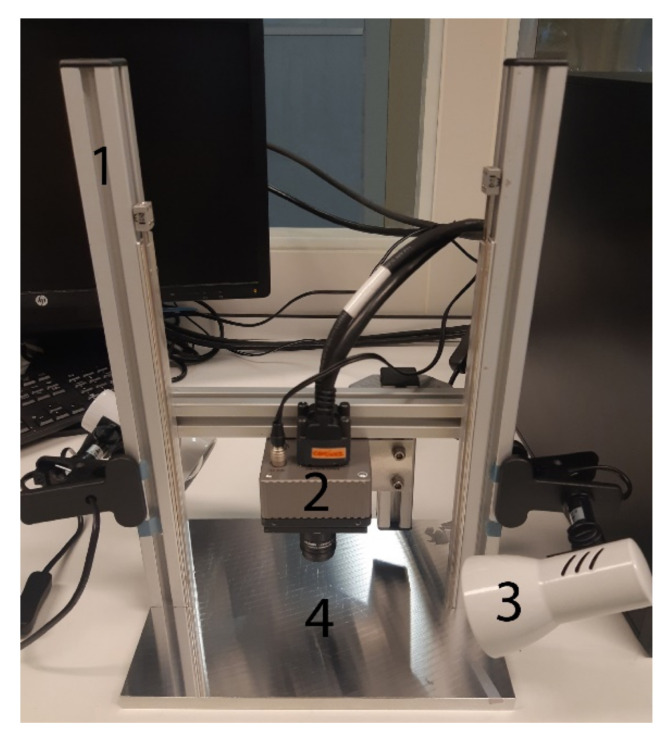
Hyperspectral data acquisition system, showing: 1. Vertical rigid stage; 2. Hyperspectral camera; 3. Halogen light source; 4. Plateau.

Images were acquired with a hyperspectral camera containing a snapshot mosaic 5 × 5 hyperspectral imaging sensor (IMEC, Leuven, Belgium). This camera can capture an entire scene containing multi-spectral images at video rate. The CMOS sensor captures 25 spectral bands with a spectral bandwidth of <15 nm in the near-infrared (NIR) range (665–975 nm). The spectral bands are arranged in a 5 × 5 mosaic grid and have a spatial resolution of 409 × 218 for each band and a total resolution of 2050 × 1080. The hyperspectral camera is controlled using a desktop PC, using the proprietary camera software. The parameters were set at a frame rate of 10 HS cubes per second, an exposure time of 10 milliseconds and a color gain of 10. The camera and two halogen light sources were mounted on a vertical framework. The halogen lights were placed under an angle of ±45° to minimize glare. In order to perform the scans, samples were placed on a black paper underneath the camera on the plateau (see Figure 1).

### 2.3. Data Acquisition

After CRS, the resected specimens of the ovaries, fallopian tubes, uterus, omentum and/or part of the intestines were sent to the pathology department. From each organ, one tissue slice of maximal 20 × 40 × 3 mm with suspected tumorous and non-tumorous tissues was taken and placed on black paper. If the specimen contained no tumor tissue, a slice containing only non-tumor tissue was taken and placed on black paper. First, a red-green-blue (RGB) image was collected with a normal camera. Thereafter a white reference image, a dark reference image and HS images of the tissue were collected with the HS camera. After the imaging procedure, the tissue samples were placed in a standard macro cassette, placed in formaldehyde and processed according to standard pathological protocols.

### 2.4. Data Pre-Processing

All hyperspectral data were pre-processed by performing image calibration, normalization by feature scaling and noise filtering, respectively.

#### 2.4.1. Image Calibration

During image calibration, the relative reflectance was calculated to correct for the signal variation between images due to non-uniform illumination and the focal plane array of the camera (pattern noise). The raw data were calibrated by using a white reference image and a dark reference image. The dark reference image was acquired by the HS system by keeping the shutter of the camera closed and was used to account for the internal noise, caused by the dark current. The white reference image was acquired by taking a picture of a white paper and was used to account for the light distribution [18,21,22]. The relative reflectance image $I_{ref}\left(x,y,\lambda \right)$ was calculated by:

(1) $$I_{ref}\left(x,y,\lambda \right)=\frac{I_{raw}\left(x,y,\lambda \right)-I_{dark}\left(x,y,\lambda \right)}{I_{white}\left(x,y,\lambda \right)-I_{dark}\left(x,y,\lambda \right)},$$

where $I_{raw}\left(x,y,\lambda \right)$ is the raw spectral image, $I_{dark}\left(x,y,\lambda \right)$ is the dark reference image, and $I_{white}\left(x,y,\lambda \right)$ is the white reference image at the sample pixel location $\left(x,y\right)$ and wavelength band λ [18].

#### 2.4.2. Normalization

The range of all features was normalized by re-scaling the range of the data to the interval $\left[min\left(x_{new}\right),\;max\left(x_{new}\right)\right]=\left[0,1\right]$ and corrected for disproportional feature contributions due to tissue morphology effects. This ensured that the classification algorithm performed a classification based on the shape of the spectral signature and not the amplitude [21]. The normalized image $\hat{x_{i}}$ was calculated by:

(2) $$\hat{x_{i}}=\frac{x_{i}-min\left(x_{i}\right)\;}{max\left(x_{i}\right)-min\left(x_{i}\right)}*\left(max\left(x_{new}\right)-min\left(x_{new}\right)\right)+min\left(x_{new}\right),$$

where$\;x_{i}$ is the relative reflectance image, $min\left(x_{i}\right)\;$the minimum value of $x_{i}$, $max\left(x_{i}\right)\;$the maximum value of $x_{i}$, $min\left(x_{new}\right)\;$the new minimum value of $x_{i}$ and $max\left(x_{new}\right)\;$the new maximum value of $x_{i}$ [23].

#### 2.4.3. Image Data Selection

HS images can contain pixels that do not contain useful spectral information for tissue classification and reduce the performance of the system, such as background pixels and glare pixels. Several images of the same sample were taken under different configurations with the hyperspectral camera. The images with the least glare were selected and included in the data set. The remaining glare pixels and background pixels were removed from the hypercube by intensity thresholding. Tissue edges of approximately 20 pixels were removed, because they were more likely to contain mixed spectra [16]. Furthermore, each hypercube was divided into a grid of 20 × 20 pixels. Within each block, the spectra were averaged to increase the robustness to registration errors and improve the classification performance [24,25].

### 2.5. Pathological Annotation

The pathological diagnosis was used as ground truth to label spectral images. A few days after the surgery, the tissue slices were processed in the standard manner for diagnostic histopathology, embedded in paraffin blocks and sectioned. The slides were stained with hematoxylin and eosin (H&E) and were then digitized. The digitized histological images of all specimens were annotated to outline tumor tissue, connective tissue, ovarian stromal tissue, fat tissue, lymphoid tissue, necrotic tissue, epithelial tissue of the intestines and muscle tissue by an experienced pathologist (P.C.E-G. or L.R.). Tissue was characterized as tumor tissue when there was >50% of tumor tissue in comparison to connective tissue. Areas which contained between 1% and 50% of tumor tissue in comparison to connective tissue were left out of the dataset.

### 2.6. Classification of Hyperspectral Data

#### 2.6.1. Image Classification

**Figure 2:**
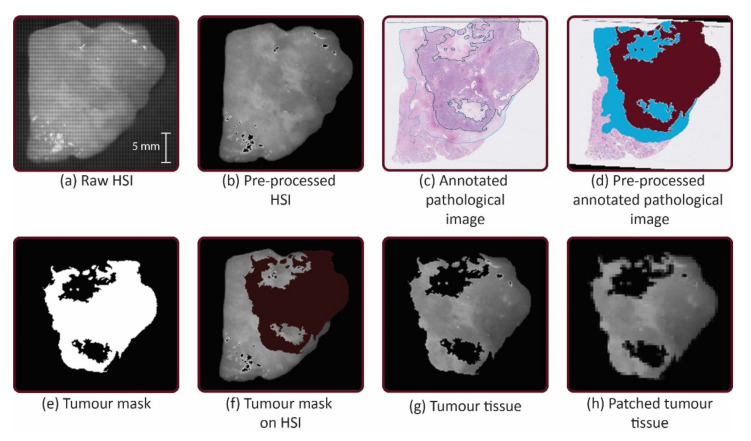
Workflow: (**a**) The hyperspectral (HS) images from the mosaic 5 × 5 hyperspectral camera were acquired after cytoreduction. (**b**) HS images were pre-processed by calibration, min-max normalization and noise filtering and transformed. (**c**) The digitized pathological images were annotated. (**d**) The annotated images were transformed. Tumor tissue was filled with the color bordeaux and the non-tumor tissue was filled with the color blue. (**e**) Tumor tissue and non-tumor tissue were selected via hue, saturation and value (HSV) color thresholding to make masks. (**f**) Selection of the tumor tissue and non-tumor tissue was made with the mask. (**g**) The HS images were multiplied by the mask to obtain tumor tissue and non-tumor tissue. (**h**) The HS images were patched into a grid of 20 × 20 pixels and features were extracted.

The annotated images and RGB image were overlaid via a non-rigid registration algorithm in MATLAB (Mathworks Inc., Natick, MA, USA, R2020b) with approximately 20 control points. This was also done with the HSI. In this way, the HSI and masks had the same configuration. The area of the tumor tissue and the area of non-tumor tissue were selected via hue, saturation and value (HSV) color thresholding to make a tumor tissue and non-tumor tissue mask. To obtain the labelled tumor and non-tumor image regions for training, the HS images were multiplied with the tumor mask and non-tumor mask, respectively. Thereafter, the HS images were patched and features were extracted (see Figure 2).

#### 2.6.2. Feature Extraction

The HS camera captures 25 images in adjacent spectral bands of 15 nm. The intensities, derivatives and intensity ratios were extracted and formed the feature set.

#### 2.6.3. Support Vector Machine Classifier

The support vector machine (SVM) classifier with linear kernel function (linear SVM) was used for our classification, as it is one of the most effective machine learning methods to classify HS data [26]. The SVM classifier finds the best hyperplane that separates the data points of tumor tissue (positive) from the non-tumor (negative) class with the largest margin between the hyperplane and any data point. For inseparable classes, the objective is the same, but the algorithm imposes a penalty on the length of the margin for every observation that is on the wrong side of its class boundary. The dataset contained significantly more non-tumor data points than tumor data points. For this reason, weights were implemented to prevent bias towards predicting the majority class and balance the dataset. The weights were assigned inversely proportionally to their frequencies.

### 2.7. Classifier Performance Evaluation

**Figure 3:**
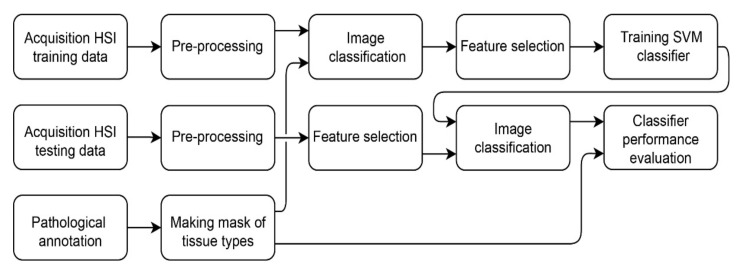
Method for training and classifying the tumor and non-tumor tissue.

The algorithm performance was validated using leave-one-out cross-validation. In order to evaluate the classification results, sensitivity, specificity, positive predictive value (PPV) and negative predictive value (NPV) based on the confusion matrix of the optimal threshold point of the receiver operating characteristic (ROC) curve were calculated. The optimal threshold point of the ROC curve was calculated using the Youden index. Since the sensitivity, specificity, PPV and NPV are dependent on the threshold value of the ROC curve, the area under the ROC curve (AUC) and the Matthews correlation coefficient (MCC) have been calculated to represent the scalar measurement for the quality of the classification [18]. The whole method is depicted in Figure 3.

## 3. Results

### 3.1. Participants and Pathologies

In total, 11 patients were enrolled in the study. Patient 5 was left out because the primary location of the tumor was uncertain. In total, 10 patients were therefore included in the study. The mean age was 58 years (range 30–78). Eight patients received three courses of chemotherapy prior to surgery (interval CRS), three patients were chemo-naive (primary CRS). The patient characteristics are provided in Table 1.

### 3.2. Spectral Signatures

**Figure 4:**
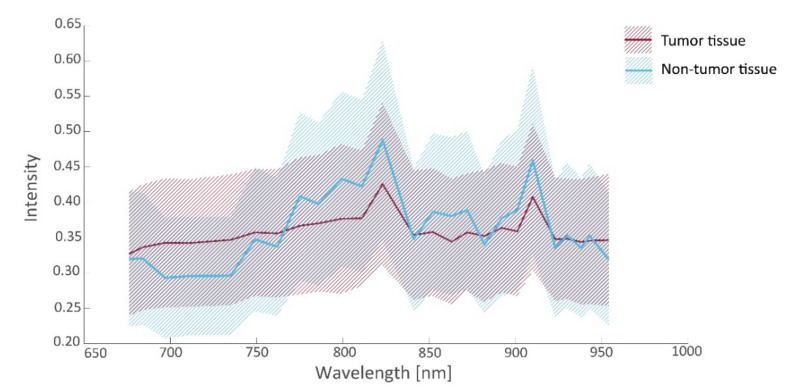
Intensity values (median and interquartile range (IQR)) as a function of wavelength for tumorous and non-tumorous tissues.

**Figure 5:**
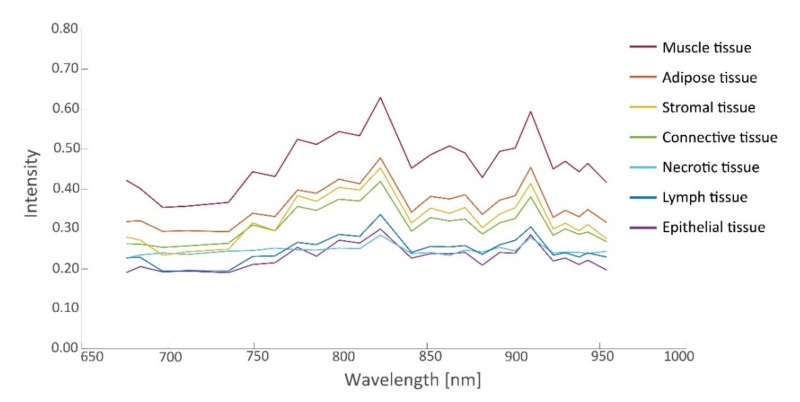
Intensity values (median) as a function of wavelength for various non-tumorous tissues.

In this study, 26 tissue samples were imaged in total, containing a total of 26,446 hyperspectral data points that were matched to histopathology, including tissue from the ovarian (13 samples), omentum (10 samples), mesenterium (1 sample) and intestines (2 samples). The amount of tumor and non-tumor data per patient are given in Table 2. The samples contained different tissue types, such as connective tissue, necrotic tissue, ovarian stromal tissue, adipose tissue, lymphoid tissue, muscle tissue, and epithelial tissue of the intestines. Figure 4 and Figure 5 present the spectral signature of the different tissue types. It should be noted that the spectral signature of tumor and non-tumor tissue show large within-class variations, which may primarily be attributed to the heterogeneity of the included tissue types. 

### 3.3. Tissue Classification

#### 3.3.1. Feature Selection

**Figure 6:**
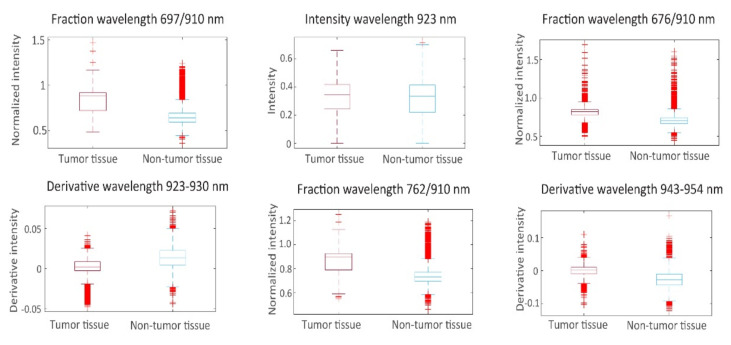
Boxplots of the first 6 principal components of prognostic spectrum features.

From the spectra visualized in Figure 4, a set of 19 prognostic features was extracted for which tumorous and non-tumorous tissues showed the largest difference. This set was composed by comparing distributions (*t*-tests) in observed mean intensities, intensity ratios, and intensity derivatives, and their influence on the AUC. The boxplots of the first six principal components of prognostic spectrum features are shown in Figure 6. The selected features include the spectral intensities at 697 nm, 775 nm, 799 nm, 823 nm, 863 nm, 872 nm, 901 nm, 910 nm, 923 nm, the intensity differences (derivatives) at 684–697 nm, 882–892 nm, 923–930 nm, 943–954 nm and the intensity ratios 676/910 nm, 697/910 nm, 762/910 nm, 872/910 nm, 923/910 nm, and 930/910 nm. Although the spectral signatures of the same tissue type vary in intensity, they run mostly in parallel. In correspondence, the interquartile range (IQR) lines in Figure 4 and Figure 5 also run parallel to the median lines. Therefore, the derivatives and in particular the fractions between intensities resulted in useful features.

#### 3.3.2. Classifier Performance 

**Figure 7:**
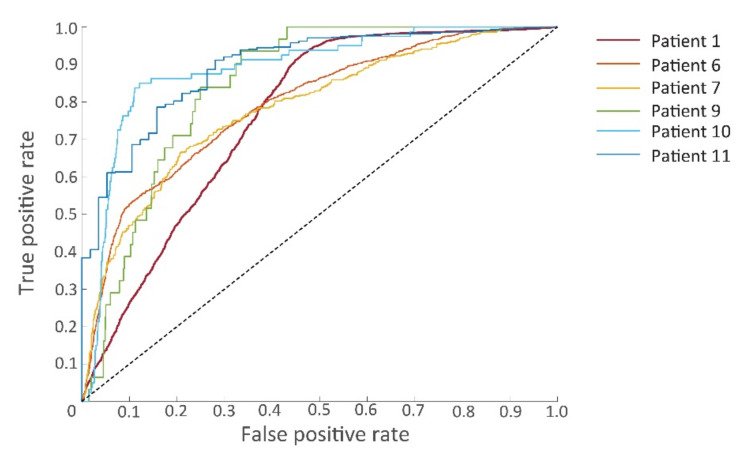
Receiver operating characteristic (ROC) curves of the six patients for whom samples contained both tumorous and non-tumorous tissues.

The prognostic features were used for training the SVM model. Classification results of tumor tissue and non-tumor tissue with the use of the linear SVM classifier are given in Table 3. The linear SVM had an overall sensitivity of 0.81, specificity of 0.75, PPV of 0.53, NPV of 0.82, AUC of 0.83 and MCC of 0.41. The ROC curves are given in Figure 7. The data set of patients 2, 3, 4, and 8 consisted only of non-tumor tissue. Without tumor tissue, several classification power metrics (sensitivity, PPV, NPV, AUC) could not be determined. 

## 4. Discussion

The most important prognostic factor for the survival of advanced-stage ovarian cancer is the completeness of CRS [5]. An intraoperative visualization technique is useful for detecting microscopic residual tumor and minimizing recurrence. In this pilot study, the feasibility of NIR HSI for the detection of malignant ovarian cancer metastases was tested ex vivo. In the current work, we looked at the difference between tumorous and healthy tissues. The linear SVM classifier had a sensitivity of 0.81, a specificity of 0.70, AUC of 0.83 and MCC of 0.41 for the detection of tumor tissue. The variations of the classification power could be explained by the non-homogeneous distribution of the tumor tissue and the types of healthy tissue across the patients, which influenced the training dataset for the SVM classifier. Furthermore, the included tissue of patient 2 was relatively small (biopsy) in comparison to the other patients (see Table 2). In future work, we aim to extend the sample size to stratify healthy tissue types and perform classification per tissue sample. 

In comparison, cryosection is used for intraoperative resection margin assessment. Cryosection has a higher classification power, but can only be applied on a small resected tissue surface outside the operating room and is time consuming [6,19,20]. Alternatively, several studies have been done using fluorescent imaging for the detection of ovarian cancer [7,8,9,10,11,27,28,29]. Promising results were achieved for EOC detection with 5-ALA (sensitivity: 0.93 specificity: 1.00 [8], sensitivity: 0.89 specificity: 1.00 [9]) and OTL38 (sensitivity: 0.84–0.98 PPV: 0.85–0.95 [11]), however fluorophore injection can lead to mild adverse side effects [8,9,10,11]. The specificity of indocyanine green is currently low (0.41–0.57) [27,28,29].

The HSI camera used has a wavelength range from 665 nm to 975 nm. In this region, several chromophores such as blood, water, melanin, fat, bilirubin, and beta-carotene contribute to the absorption of light and the spectral signatures of tissue [13]. An advantage of the NIR wavelength range of the camera is that it can measure up to several millimeters and is scattering dominant for biological tissue, which leads to more detectable differences between tissue types [14,19]. The visual wavelength range for the detection of tumor tissue is also evaluated in the study of Baltussen et al. [30] and Kho et al. [16]. Both studies found that an increased spectral range (visual and NIR) had the best result. However, for in vivo studies, the visual wavelength range (400–700 nm) is scattering dominant for hemoglobin, resulting mostly in the visualization of blood and not tumor tissue [13,30]. Beaulieu et al. [31] also looked at SWIR wavelengths (900–2500 nm) and found that there may be tumor-specific signals in this range. According to Halicek et al. [32], the SWIR wavelength has scattering dominants for water and adipose tissue. This can be of added value for the detection of tumor tissue in adipose regions such as the omentum.

Several studies have tried to compare different machine learning classifiers such as artificial neural networks and LDA for the detection of cancer [13,15,16,17,18,19,20,21,31,32]. Artificial neural network algorithms show promising results [21,32]. However, they can be prone to overfitting if there is insufficient data, and the results can be more difficult to interpret [12]. For the classification of ovarian cancer, the determination of an optimal classifier will be valuable in future work. 

The tissue annotated as tumor also included small amounts of connective tissue, calcification and other types of non-tumor tissue. This decreases the performance of the classifier. In this study, tumorous tissue was defined as regions where the cellular content contains more than 50% of tumor cells. In the future, studies should be extended to include areas of tissue with less than 50% of tumor cells. Furthermore, there was a discrepancy between the measured depth of the HS camera and the pathological ground truth. The HS camera can evaluate up to a few millimeters deep, but the H&E section only provides information on the superficial cell layers of the slice [14,16,20]. In addition, for the first four patients, the bottom of the tissue slide instead of the top of the tissue slide was inspected (sample thickness: 3 mm), due to the wrong configuration of the tissue in the pathological tray.

Finally, registration errors could occur when the histopathological slide and HS image do not have an exact match in shape, e.g., due to deformations during the tissue handling. Furthermore, other parts of the image and spectral data processing may still be improved, e.g., improving image registration to cope with elastic deformation of the tissue. The feature extraction step may be improved using a principal component analysis (PCA) or other feature selection methods [33]. This method will result in a minimal and independent set of features to describe the data.

This study shows the feasibility of detecting tumor tissue with HSI and several steps to optimize the current methodology. To bring HSI into clinical practice and reduce the recurrence of EOC, additional research is needed. Future improvements could consist of the optimization of the classification algorithm, stratification of tissue types and in vivo studies to assess the tumor margin intraoperatively.

## 5. Conclusions

Although in vivo studies are needed, we exploited HSI for ovarian cancer detection, paving the way for improving surgical outcome and patient prognosis. Our pilot study shows that HSI is a promising technique for detecting tumors. In this study, a sensitivity of 0.81, a specificity of 0.70, AUC of 0.83 and MCC of 0.41 was achieved for the detection of tumor tissue ex vivo. Although cryosection still performs more accurately, HSI can scan larger areas, is faster, non-contact and non-invasive and can be used inside the operating room. Compared to fluorescent imaging, HSI does not require the intake of fluorophores and does not result in the related adverse side effects.

## Figures and Tables

**Table 1:** Patient characteristics.

Patient Number	Primary Location	Histology	Grade	FIGO Stage	Procedure	Tissue Type
1	Ovarian	Serous adenocarcinoma	3	IIIC	PDS ^a^	A: Ovarian B: Ovarian C: Ovarian D: Omentum
2	Ovarian	Serous carcinoma	3	IV	IDS ^b^	A: Mesenterium
3	Ovarian	Serous adenocarcinoma	1/3	IV	IDS ^b^	A: Omentum B: Ovarian
4	Ovarian	Serous adenocarcinoma	3	IV	IDS ^b^	A: Omentum B: Omentum C: Intestines
5		Mucinous adenocarcinoma	3	IV	PDS ^a^	A: Omentum
6	Ovarian	Serous adenocarcinoma	3	IIIC	IDS ^b^	A: Ovarian B: Ovarian C: Intestines D: Omentum E: Omentum
7	Ovarian	Serous adenocarcinoma	3	IV	IDS ^b^	A: Omentum B: Ovarian C: Ovarian
8	Ovarian	Serous adenocarcinoma	3	IIIC	IDS ^b^	A: Omentum B: Ovarian
9	Ovarian	Serous adenocarcinoma	3	IV	IDS ^b^	A: Ovarian B: Ovarian
10	Ovarian	Serous adenocarcinoma	3	IV	IDS ^b^	A: Ovarian B: Omentum C: Ovarian
11	Ovarian	Serous adenocarcinoma	1	IV	PDS ^a^	A: Omentum

^a^—PDS: Primary debulking surgery (chemo-naïve); ^b^—IDS: Interval debulking surgery (received prior to chemotherapy).

**Table 2:** Amount of tumor and non-tumor data points per patient.

Patient	1	2	3	4	6	7	8	9	10	11
Total	7819	123	1678	3134	7102	1236	1663	1564	1382	745
Tumor	5065	0	0	0	1012	440	0	31	80	688
Non-tumor	2754	123	1678	3134	6090	796	1663	1533	1302	57

**Table 3:** Classification result of leave-one-out cross-validation.

Patient	Sensitivity	Specificity	PPV ^a.^	NPV ^b.^	AUC ^c.^	MCC ^d.^
1	0.91	0.55	0.79	0.77	0.76	0.51
2	-	0.00	0 *	-	-	-
3	-	0.55	0 *	1.00 *	-	-
4	-	0.99	0 *	1.00 *	-	-
6	0.55	0.87	0.42	0.92	0.79	0.38
7	0.66	0.79	0.64	0.81	0.78	0.45
8	-	1.00	0 *	1.00 *		
9	0.95	0.67	0.05	1.00	0.84	0.18
10	0.85	0.88	0.30	0.99	0.89	0.46
11	0.91	0.72	0.98	0.40	0.89	0.49
Mean	0.81	0.70	0.53	0.82	0.83	0.41

^a^—PPV: Positive predictive value; ^b^—NPV: Negative predictive value; ^c^—AUC: Area under the curve; ^d^—MCC: Matthew’s correlation coefficient; * Samples without tumor tissue. PPV and NPV were not included in calculation of the mean.

## Data Availability

All data can be found in the text.
